# DCJ-RNA - double cut and join for RNA secondary structures

**DOI:** 10.1186/s12859-017-1830-6

**Published:** 2017-10-16

**Authors:** Ghada H. Badr, Haifa A. Al-aqel

**Affiliations:** 1 IRI- The City of Scientific Research and Technological Applications, University and Research District, P. O. 21934, New Borg Alarab, Alexandria, Egypt; 2 University of Ottawa, Faculty of Engineering, Ottawa, Canada; 3 Imam Mohammad ibn Saud Islamic University, College of Computer and Information Sciences, Riyadh, Saudi Arabia

**Keywords:** Genome Rearrangement, RNA Secondary Structure, DCJ, Similarity Measure, Sorting Scenario

## Abstract

**Background:**

Genome rearrangements are essential processes for evolution and are responsible for existing varieties of genome architectures. Many studies have been conducted to obtain an algorithm that identifies the minimum number of inversions that are necessary to transform one genome into another; this allows for genome sequence representation in polynomial time. Studies have not been conducted on the topic of rearranging a genome when it is represented as a secondary structure. Unlike sequences, the secondary structure preserves the functionality of the genome. Sequences can be different, but they all share the same structure and, therefore, the same functionality.

**Results:**

This paper proposes a double cut and join for RNA secondary structures (DCJ-RNA) algorithm. This algorithm allows for the description of evolutionary scenarios that are based on secondary structures rather than sequences. The main aim of this paper is to suggest an efficient algorithm that can help researchers compare two ribonucleic acid (RNA) secondary structures based on rearrangement operations. The results, which are based on real datasets, show that the algorithm is able to count the minimum number of rearrangement operations, as well as to report an optimum scenario that can increase the similarity between the two structures.

**Conclusion:**

The algorithm calculates the distance between structures and reports a scenario based on the minimum rearrangement operations required to make the given structure similar to the other. DCJ-RNA can also be used to measure the distance between the two structures. This can help identify the common functionalities between different species.

## Background

DNA is a biological blueprint that a living organism must have to exist and remain functional. RNA holds the guidelines for this blueprint. RNA is responsible for transferring the genetic code from the nucleus to the ribosome to build proteins. It is identified as a series of letters with bases {A, C, G, U}. RNA’s secondary structure is required to define the functionality of RNA molecules. In contrast to representing the genome as a sequence, representing it as a secondary structure provides more insight into the genome’s function. In this paper, RNA’s secondary structure is presented using a component-based representation, which was recently proposed in 2011 [1]. In contrast to similarity between gene orders, identifying the similarity of functioning between two structures has a greater impact on comparing species. Comparing two species based on their secondary structures provides more information and reveals more accurate evolutionary scenarios [2]. Comparison of two species based on their secondary structures can also be combined with existing sequence-based algorithms to enhance sequence-based algorithms efficiency [3]. This helps create more accurate phylogenies [4].

The paper outline is as follows - the RNA secondary structure is presented using a component-based representation. The researchers proceed to describe the measures that are used to determine the similarity between components of the given structures. Genome rearrangement in terms of sequences and its operations, sorting scenario, and distance measures are summarized. We then propose a DCJ-RNA rearrangement algorithm and explain it in detail. Two case studies using real data are presented, illustrating the detection and application of the proposed rearrangement operations for real RNA secondary structures. The results demonstrate that the proposed algorithm provides one evolutionary scenario that shows how to alter one structure to make it similar to the other or the same as the other. Preliminary work has been presented as a poster in [5].‬‬‬‬‬‬‬‬‬‬‬‬

### RNA secondary structure component-based representation

**Fig. 1:**
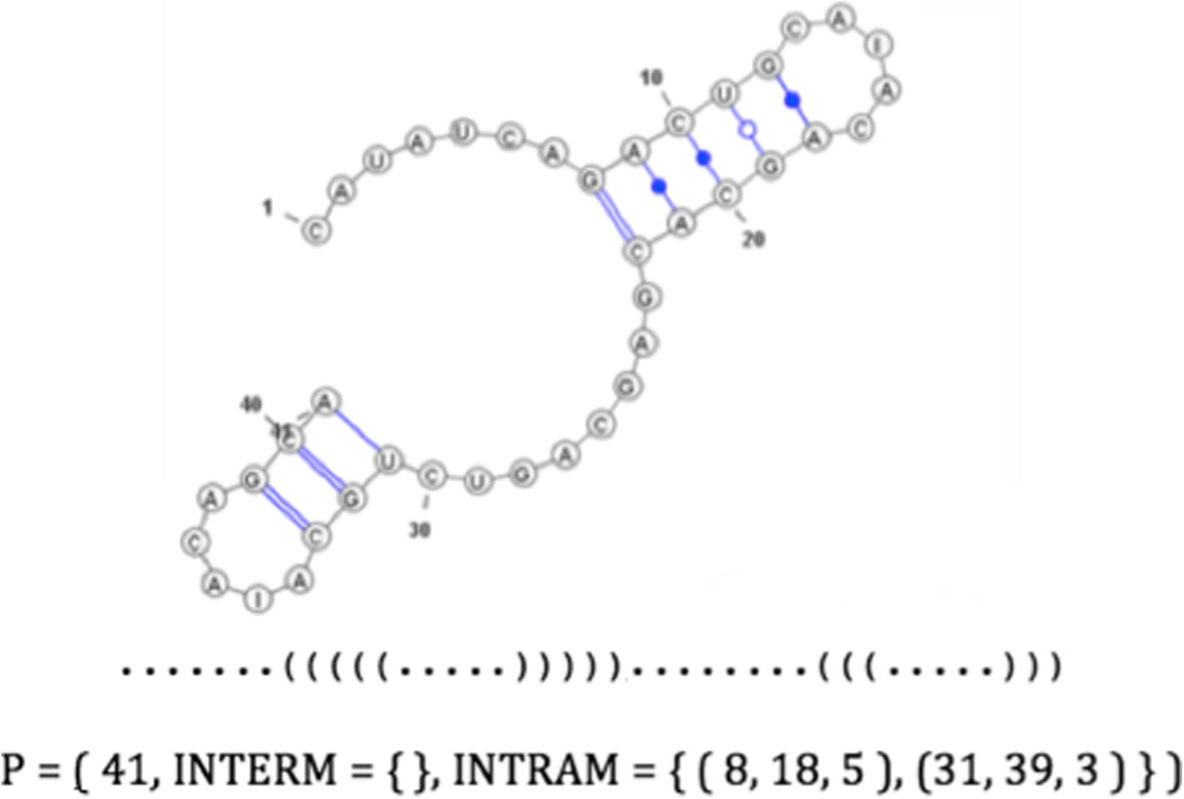
An example of a component-based representation

Badr and Turcotte [1] propose a component-based structure to define interacting and non-interacting patterns as follows - the representation can be used to define interacting and non-interacting patterns for RNA secondary structures. A pattern (P = {p_1_, p_2_. .. p_m_}) is defined by its sub-patterns (P_i_, 0 < i < m). Each sub-pattern is defined by its length and intermolecular (INTERM) and intramolecular (INTRAM) components. For non-interacting patterns, there are no INTERM components. These components are defined by their opening bracket (OB), closing bracket (CB), length, and relative locations within the sub-patterns. In the INTERM component, OB and CB are located in two different sub-patterns. In the INTRAM component, OB and CB are located in the same sub-pattern. In the INTERM component, OB and CB must be in different sub-patterns, which suggests that there must be at least two sub-patterns to have INTERM components. OB is located in p_i_, and CB is located in another sub-pattern (p_j_), where j > i and 1 ≤ j ≤ m. OB and CB are defined by their lengths and locations relative to the beginning of p_i_. Thus, INTERM = {OB, CB, j, len}. In INTRAM components, OB and CB have to be in the same sub-pattern, which indicates that there must be at least one sub-pattern to have INTRAM components. OB and CB are located in p_i_, where 1 ≤ i ≤ m. OB and CB both are defined by their location and length. Therefore, INTRAM = {OB, CB, len}. Figure 1 shows an example of a non-interacting pattern.

### Similarities between two RNA secondary structures (Alignment distance)

Badr and AlTurki [6] propose a similarity measure based on aligning two secondary structures that are presented using a component-based representation. The algorithm extracts the features of each component, which are OB, CB, and length. The similarity between two structures depends on the component’s position, full length, and stem length. These measures are used in the new proposed algorithm. The equations that are applied to calculate the similarity between two components, a_i_ in structure A and b_j_ in structure B, d(f_ai_, f_bj_), can be found in [6]. The similarity measure between two components is used to calculate the dynamic programming matrix using the method proposed by Needleman and Wunsch [7]. The alignment score between two structures is calculated using Eq. 1, while the percentage of the similarity between two structures is calculated using Eq. 2 [6].

1 $$ Score\left(a,b\right)=\left\{{\sum}_{i=1}^n{\sum}_{j=1}^m\begin{array}{c}d\left( fai, fbj\right)\kern1.25em if\ {a}_i\  is aligned with\ {b}_j\\ {}0\  otherwise\end{array}\right\} $$

2 $$ \mathrm{Score}\  \mathrm{percentage}\ \left(\mathrm{a},\mathrm{b}\right)=\frac{\mathrm{Score}\left(\mathrm{a},\mathrm{b}\right)}{\operatorname{Max}\left(\mathrm{a},\mathrm{b}\right)} $$

where Max(*a*, *b*) = Max {*Score*(*a*, *a*), *Score*(*b*, *b*.)}

RSmatch [8], which is another alignment distance, is a tool for aligning RNA secondary structures and is also used for motif detection. Determined with widely used algorithms for RNA folding, it decomposes the secondary structure of RNA into a set of atomic structural components. These components are further organized using a tree model to capture the structural particularities. RSmatch can find the optimal global or local alignment between two RNA secondary structures using two scoring matrices - one for single-stranded regions and the other for double-stranded regions. Jiang et al. [9] define the alignment of trees as a measure of similarity between two secondary structures in tree representation.

### Sequence-based genome rearrangements

Genomes can be modeled using permutations. Each gene can be allocated once at the genome and assigned a unique number. A gene is modeled by a signed integer when the gene strand is known to biologists [10, 11].

#### Rearrangement operations

Two genomes can have the same number of genes but may have different orders. A sequence of operations can be applied to change one genome into another. The most common rearrangement events or operations are as follows [12, 13]:

- Inversion - This reverses the orientation of a gene (or a group of genes).
- Transposition - This changes the order of a gene (or a group of genes). In other words, if the gene is located in one index, it is moved to another index.
- Gain - This adds a gene (or a group of genes) to a genome.
- Loss - This removes a gene (or a group of genes) from a genome.
- Duplication - This duplicates a specific gene (or a group of genes) within a genome.

#### Distance measures

The distance between two genomes is the minimum number of events or operations that are required to transform one genome into the other. Yancopoulos et al. [14] first proposed double cut and join (DCJ) operations. A DCJ operation consists of cutting a genome at two distinct positions and joining the four resulting open ends in a different way. Since a gene (e.g., a) has an orientation, its two ends, namely the extremities, can be distinguished and denoted as at (tail) and ah (head). An adjacency in a genome is either the extremity of a gene that is adjacent to one of its telomeres or a pair of consecutive gene extremities in one of its chromosomes.

DCJ distance consists of two operations - cut, which cuts an adjacency in two telomeres, and join, which connect two telomeres to form an adjacency. A model in which any operation consists of two cuts followed by two joins on the extremities is considered a DCJ operation [15]. DCJ allows for multi-chromosomal genomes with both circular and linear chromosomes.

DCJ distance can be easily calculated with the assistance of an adjacency graph, which is a two-part multigraph in which each partition corresponds to the set of adjacencies of one of the two input genomes. An edge connects the same extremities of genes in both genomes. In other words, a one-to-one correspondence exists between the set of edges in an adjacency graph and the set of gene extremities. Vertices have degree one or two. Therefore, an adjacency graph is a collection of paths and cycles. DCJ distance can be define as follows:

3 $$ \mathrm{dDCJ}\ \left({\mathrm{G}}_1,{\mathrm{G}}_2\right)=\mathrm{N}-\left(\mathrm{c}\left({\mathrm{G}}_1,{\mathrm{G}}_2\right)+\mathrm{p}\left({\mathrm{G}}_1,{\mathrm{G}}_2\right)/2\right) $$

In this equation, c (G_1_, G_2_) is the number of cycles, and p (G_1_, G_2_) is the number of odd paths in the adjacency graph.

#### Sorting scenario

One related issue is identifying a sorting scenario for the given distance, which provides the operations themselves. A single or number of possible solutions or sorting sequences can be found.

Bergeron et al. [11] provide an algorithm to obtain the DCJ operation in O(n) time (Algorithm 1). Mathematically, sorting using DCJ operations is simple. As with DCJ distance, DCJ operations take two adjacencies or telomeres, cut the adjacencies/telomeres, and create new adjacencies or telomeres. There are several DCJ operation types. A DCJ operation may create two adjacencies by cutting two adjacencies. A DCJ operation may also create an adjacency and telomere by cutting an adjacency and removing a telomere. In addition, a DCJ operation can consist of forming two telomeres by cutting an adjacency. Finally, DCJ operations may create an adjacency by removing two telomeres.
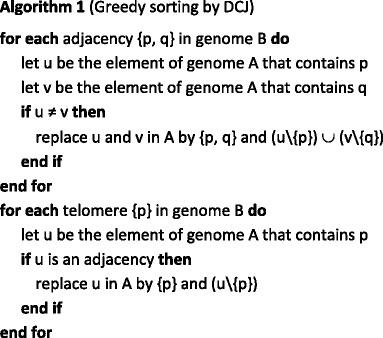


## Method: DCJ-RNA algorithm

The RNA component-based rearrangement algorithm uses a component-based representation [2] that allows for the unique description of any RNA pattern and shows the main features of the pattern efficiently. The proposed algorithm also uses the DCJ algorithm to describe rearrangement operations. It uses classical operations (inversions, translocations, fissions, fusions, transposition, and block interchanges) with a single operation and provides multi-chromosomal genomes. The DCJ-RNA algorithm (Algorithm 2) is described next.
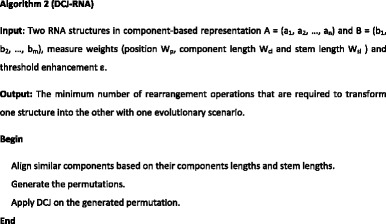


The DCJ-RNA algorithm completes three main steps:

- **Step 1 -** Alignment of similar components based on their component lengths and stem lengths.

In this step, calculate the similarity between components in terms of their component lengths and stem lengths [6]. Similar components are assigned together, beginning with those with the greatest similarity. The similarity measure that is used in this step is as follows -

4 $$ {\mathrm{d}}_1\left({\mathrm{f}}_{\mathrm{ai}},{\mathrm{f}}_{\mathrm{bj}}\right)=\mathrm{ComponentLength}\left({\mathrm{f}}_{\mathrm{ai}},{\mathrm{f}}_{\mathrm{bi}}\right).\mathrm{StemLength}\left({\mathrm{f}}_{\mathrm{ai}},{\mathrm{f}}_{\mathrm{bi}}\right) $$

Then, a matrix (m × n) is built; the entries are the component similarities in terms of component length and stem length. The rows represent the components of the first structure, and the columns represent the components of the second structure. We then search for the maximum entry (greedy) in the matrix. If it is greater than the threshold enhancement (ε) (the minimum similarity score between two components), the components are assigned together, and the corresponding row and column are deleted. If maximum similarity appears in more than one entry, the position similarity is compared between those components only and the assigned components with the greatest similarity in position. Table 1 shows the matrix structure.

- **Step 2 -** Permutation generation

**Table 1:** Component length and stem length similarity

	a_1_	a_2_	a_3_	..	a_n_
b_1_					
b_2_					
b_3_					
b_m_					

In this step, a corresponding permutation is generated for each of the two structures. This is completed by determining the components to be inserted or deleted, as well as the order of the similar components using the alignment that is generated from step 1. A two-dimensional array of 3 Χ in size (the maximum number of components in A or B + 1) is constructed and identified as SortArray. The first row contains the desired structure, the second row contains the deleted components from the actual structure, and the third row contains the inserted components from the desired structure. An index value of zero for the first row is reserved for the number of components in the actual structure. An index value of zero for the second row is reserved for the number of deleted components. For third row, an index of zero is reserved for the number of components. Table 2 shows the SortArray structure.

- **Step 3 -** Applying the DCJ algorithm.

**Table 2:** The structure of SortArray

Index	0	1	2	3	4	5	…	Max + 1
SortArray[0]	# of components in actual structure	Desired Structure Components						
SortArray[1]	# of deleted components	Deleted Components						
SortArray[2]	# of inserted components	Inserted Components						

The component numbers are used to determine the permutations in the DCJ algorithm [16]. Two permutations are provided. The first is for the given or actual permutation, and the second permutation is for the desired one.

Each permutation has two chromosomes -

- **For the first permutation -** The first chromosome is the actual structure of the components, and the second chromosome is the inserted components.
- **For the second permutation -** The first chromosome is the desired structure, and the second chromosome consists of the deleted components.

Each permutation is represented by its adjacencies and telomeres. Finally, the DCJ algorithm is applied to the first and second permutations as input.

The DCJ algorithm [17] is modified in the way that it is applied to sort the first chromosome from the second permutation; this changes the first chromosome of the first permutation. The second chromosome of the second permutation consists of the deleted components, which do not need to be sorted.

### Example

**Fig. 2:**
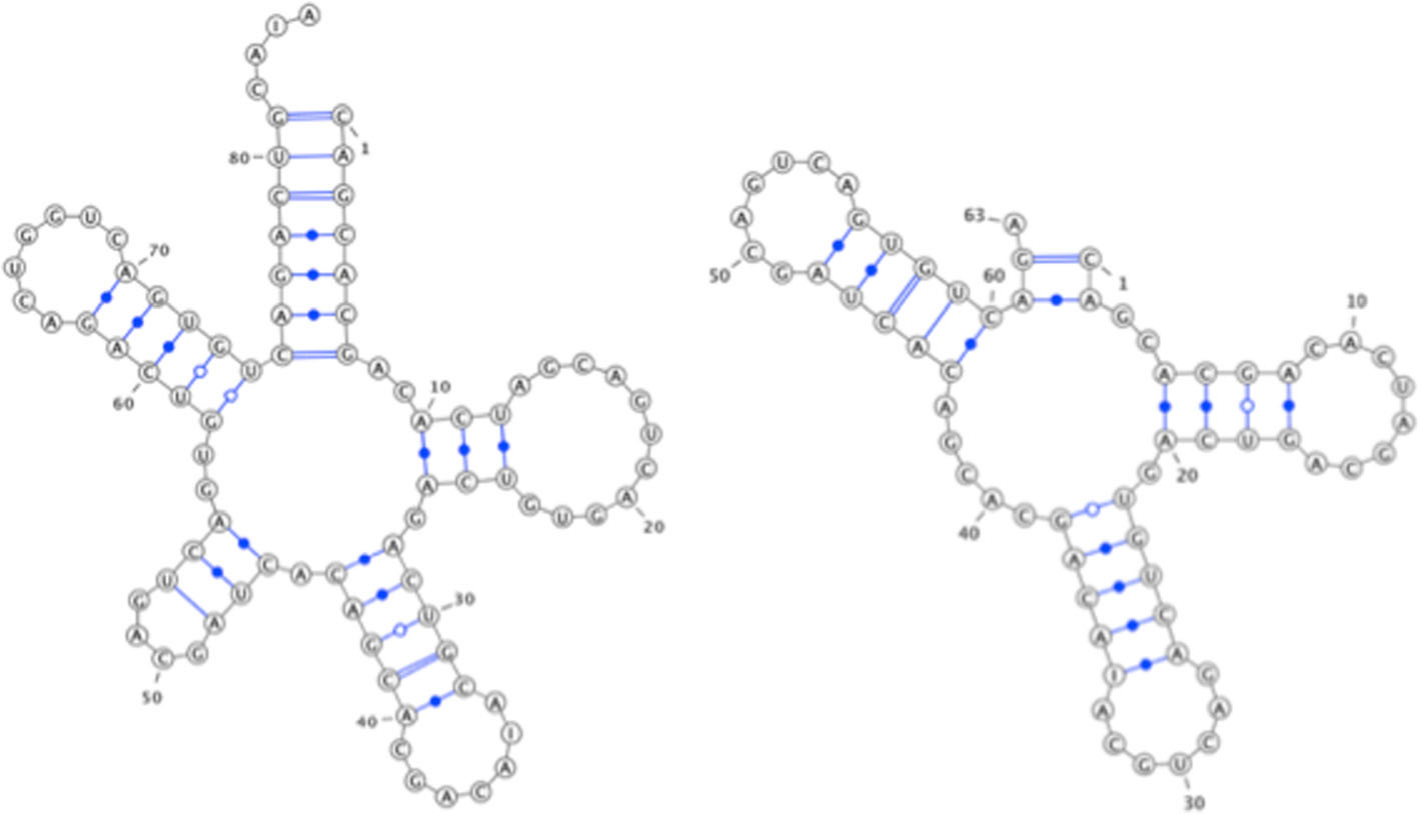
Structure A (left) and structure B (right)

In order to clarify the steps of the algorithm, real RNA secondary structures from the Genomic tRNA Database [18] are used as examples. The first structure is for *E. coli* tRNA for leucine (A), while the other structure is for *E. coli* tRNA for alanine (B) (see Fig. 2).

The two structures are presented using a component-based representation -

- A = (85, INTERM = {}, INRAM = {a_1_ = (1, 75, 7), a_2_ = (10, 24, 3), a_3_ = (28, 40, 5), a_4_ = (46, 53, 3), a_5_ = (58, 70, 5)})
- B = (76, INTERM = {}, INTRAM = {b_1_ = (1, 66, 7), b_2_ = (10, 22, 4), b_3_ = (27, 39, 5), b_4_ = (49, 61, 5)})
- The measure weights are equal to one, and threshold enhancement (ε) is equal to 0.5.

- **Step 1 -** Alignment of similar components based on their component lengths and stem lengths.

In this step, the similarity between components is calculated in terms of their component lengths and stem lengths. Similar components are assigned together, beginning with those with the greatest similarity (greedy).

In this example, the similarity between components is shown in the matrix in Table 3. First, the maximum number is one. The components are assigned together, and the row and column are removed. In this case, d_1_ (a_3_, b_3_) and d_1_ (a_3_, b_4_) are at the same position, so the nearest components are assigned in terms of their position (a_3_ and b_3_). The same case applies for d_1_ (a_5_, b_3_) and d_1_ (a_5_, b_4_). The maximum value, which is 0.83, is searched for once again. Then, a_2_ and b_2_ are assigned, and the row and column are deleted. The next value is 0.39, which is less than the threshold enhancement (ε) value, suggesting that b_1_ must be inserted and that a_1_ must be deleted. Then, a_4_ is deleted because no other components remain from the second structure.

- **Step 2 -** Permutation generation

**Table 3:** Similarity between components based on component length and stem length

	b_1_	b_2_	b_3_	b_4_
a_1_	0.39	0.24	0.29	0.29
a_2_	0.34	0.83	0.75	0.75
a_3_	0.25	0.86	1	1
a_4_	0.22	0.66	0.56	0.56
a_5_	0.25	0.86	1	1

In this step, similar components are mapped according to the process outlined in the previous step. The inserted components and deleted components are then identified (Table 4).

- **Step 3 -** Applying the DCJ algorithm.

**Table 4:** SortArray for the example

Index	0	1	2	3	4	5
SortArray[0]	5	6(b_1_)	2(a_2_)	3(a_3_)	5(a_5_)	
SortArray[1]	2	1(a_1_)	4(a_4_)			
SortArray[2]	1	6(b_1_)				

The permutations are constructed to apply the DCJ algorithm. The first permutation is chr_1_ = {1, 2, 3, 4, 5} and chr_2_ = {6}. The permutations are represented as a sequence of numbers. To differentiate between the components of the first structure and the second one, the researchers represent the second structure’s component i as i + N, where N equals the number of components in the first structure. The second permutation is chr_1_ = {6, 2, 3, 5} and chr_2_ = {1, 4}.

Then, each genome is represented with its adjacencies and telomeres to ensure that the DCJ algorithm can be applied; the first and second permutations are as follows:

- The first permutation is: {{1 t}, {1 h, 2 t}, {2 h, 3 t}, {3 h, 4 t}, {4 h, 5 t}, {5 h}, {6 t}, {6 h}}
- The Second permutation is: {{6 t}, {6 h, 2 t}, {2 h, 3 t}, {3 h, 4 t}, {4 h, 5 t}, {5 h}, {1 t}, {1 h, 4 t}, {4 h}}

In addition, {1 t}, {1 h, 4 t}, and {4 h} will not be sorted because they are included in the second chromosome. After applying the DCJ algorithm, the number of DCJ operations (3) is retrieved, as well as the sorting scenario is:

- {{{6 t}, {1 h, 2 t}, {1 t}, {2 h, 3 t}, {3 h, 4 t}, {4 h, 5 t}, {5 h}, {6 h}},
- {{6 t}, {6 h, 2 t}, {1 h}, {1 t}, {2 h, 3 t}, {3 h, 4 t}, {4 h, 5 t}, {5 h}},
- {{6 t}, {6 h, 2 t}, {1 h}, {1 t}, {2 h, 3 t}, {3 h, 5 t}, {4 h, 4 t}, {5 h}}}.

**Fig. 3:**
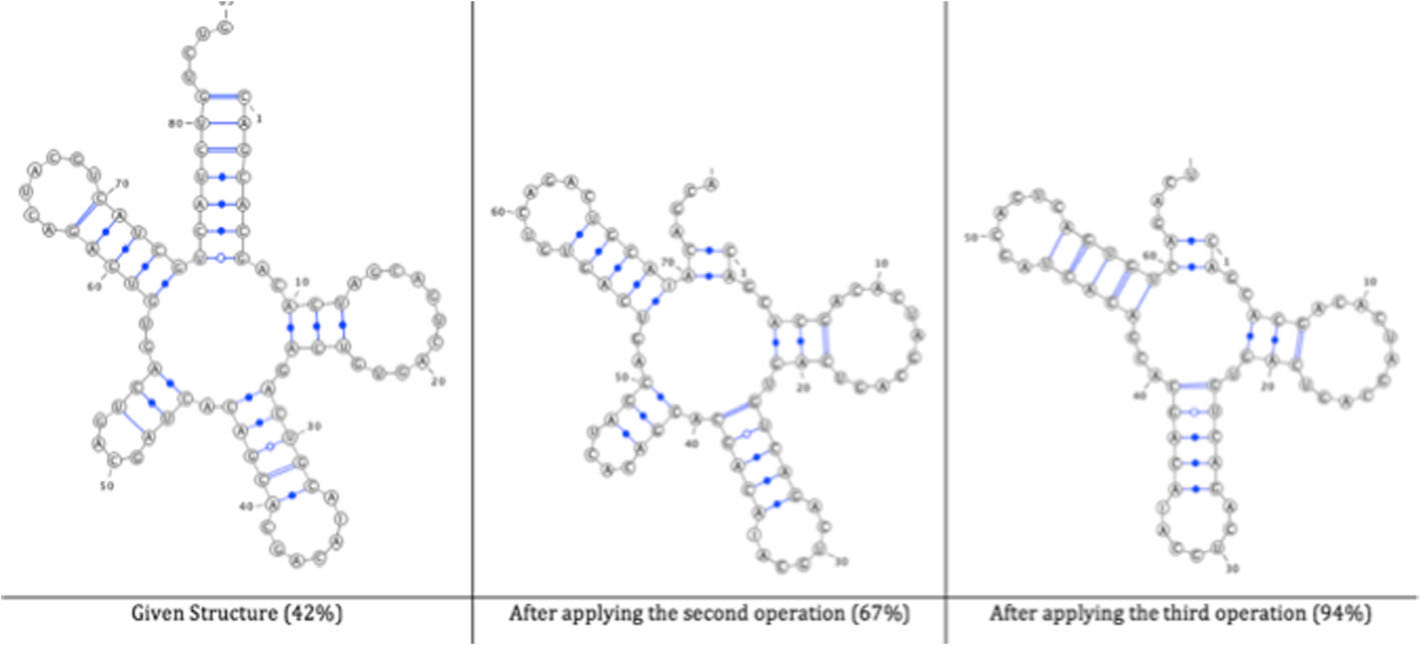
The given structures following each operation

Figure 3 shows the given structures following each rearrangement operation, as well as the similarity score with the original structure after applying each rearrangement operation. It also shows the final desired operation.

**Fig. 4:**
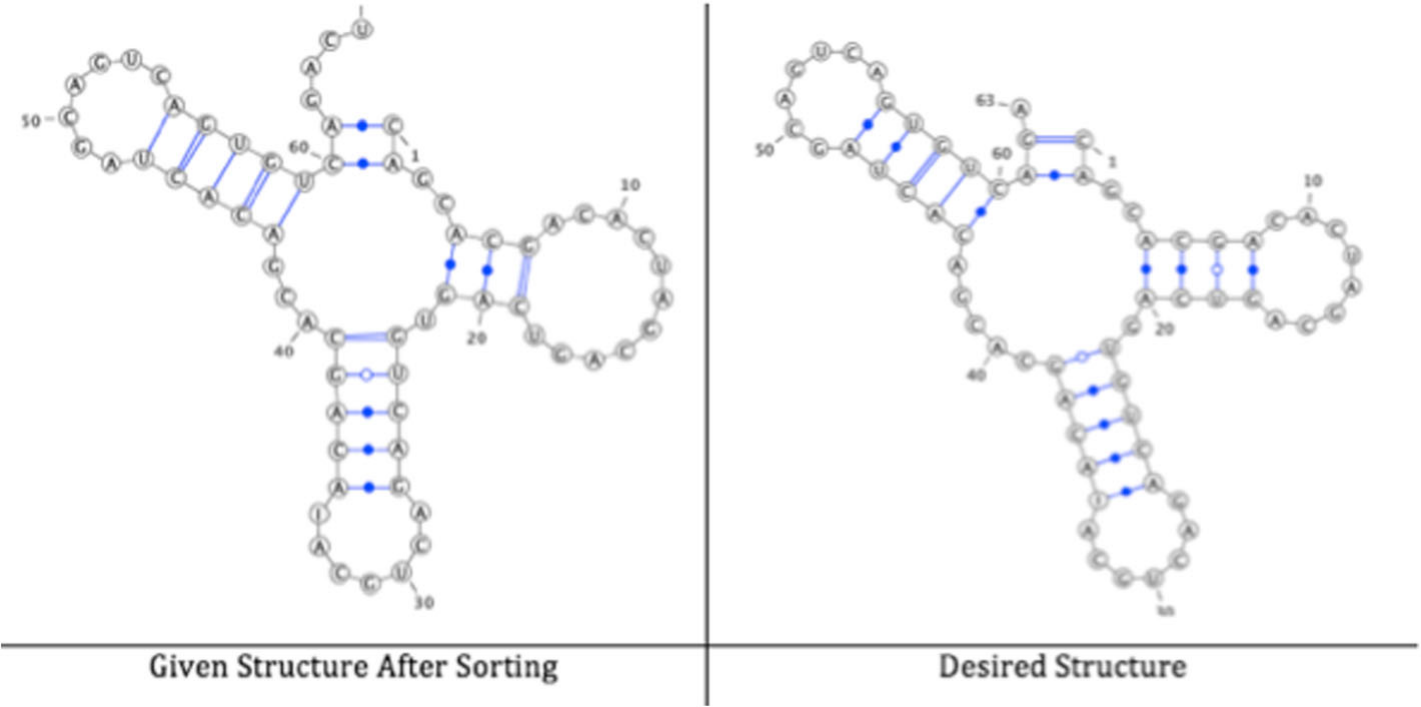
Structure A after applying the DCJ-RNA algorithm

To demonstrate the effect of the DCJ-RNA on increasing the similarity between the structures, the CompPSA algorithm [6] is used to calculate the similarity between the structures before and after applying the algorithm. The similarity between the structures is 42% before applying any changes and increases to 94% after applying the DCJ-RNA algorithm (Fig. 4).

## Results and discussion

To test and validate the DCJ-RNA algorithm, extensive experiments are conducted, three experiments are applied to three different datasets.

### Datasets

There are three different datasets - adjust dataset, accuracy dataset and scalability dataset. In this section, each dataset is described in detail.

#### Adjust dataset

**Fig. 5:**
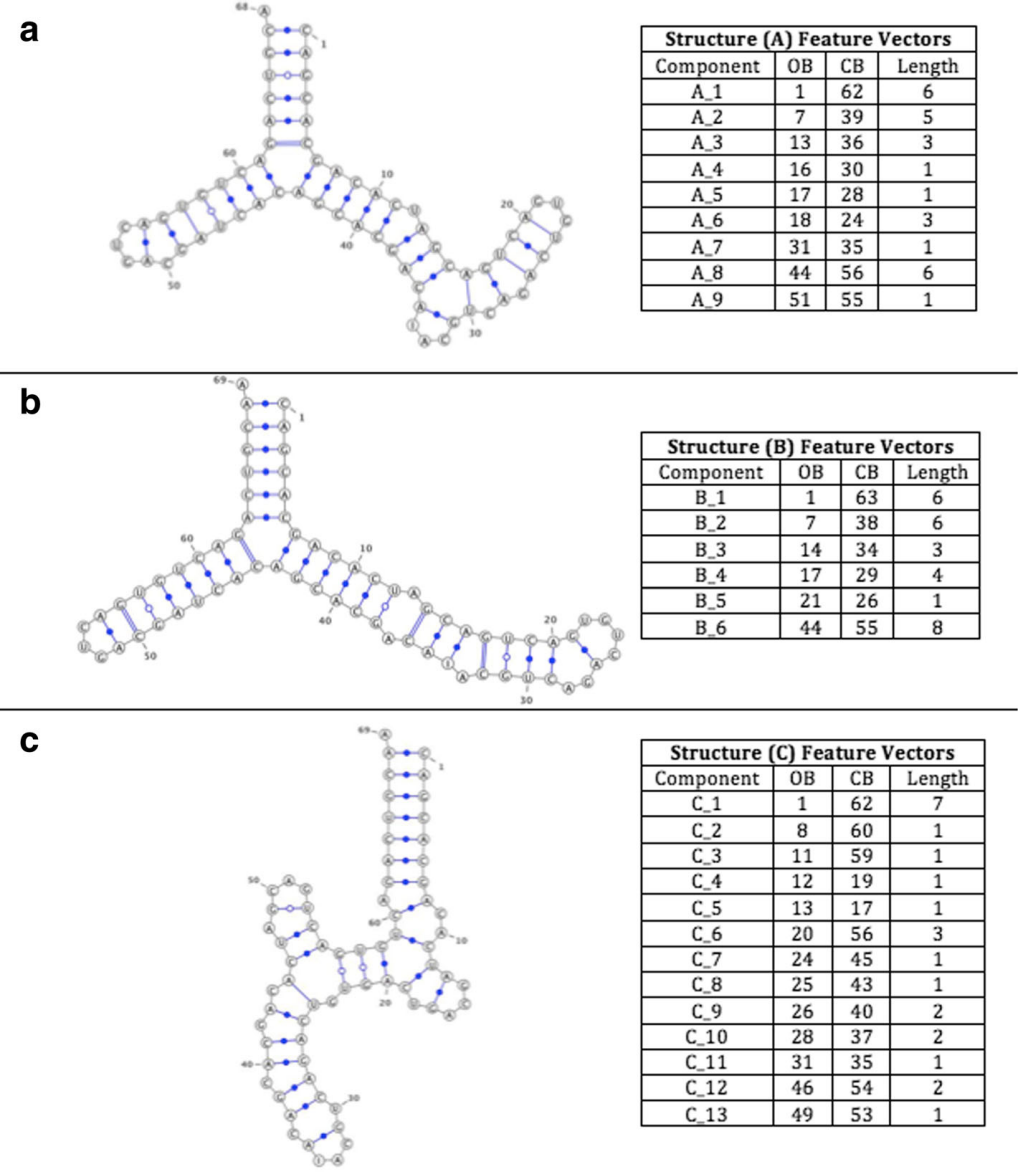
Structures A, B, and C, respectively, with their features listed as follows (ComponentID, opening bracket, closing bracket, component length)

This dataset consists of three real RNA structures named A, B and C shown in Fig. 5 where selected from the NCBI GenBank [16]. it is used to determine the best threshold enhancement (ε) value. There are two cases for RNA similarities. Dissimilar sequences and exact/approximate similar structures, structures A and B are used. In other case, dissimilar structures and exact/approximate similar sequences, structures A and C are used.

#### Accuracy dataset

**Fig. 6:**
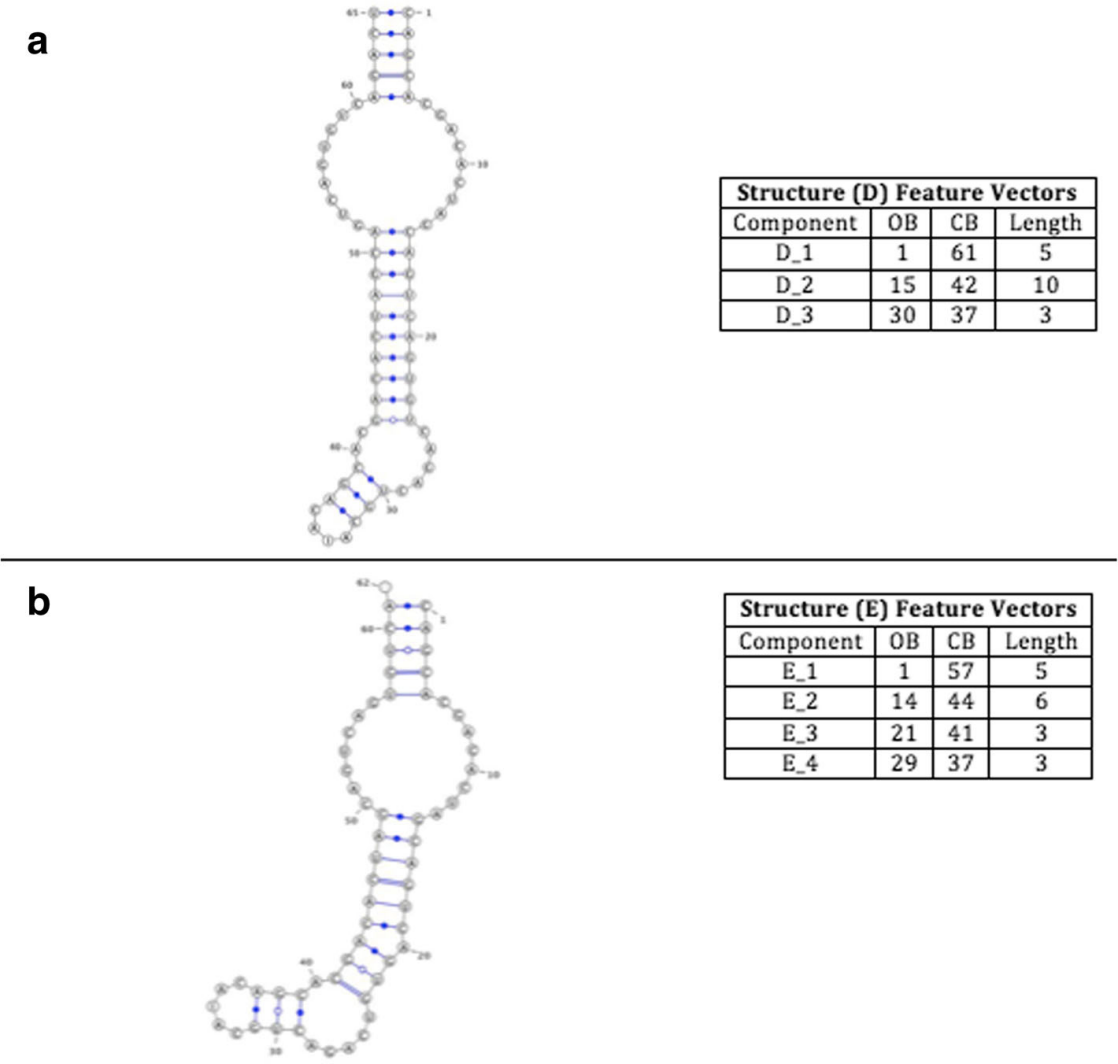
Structures D and E, respectively, with their features listed as follows (ComponentID, opening bracket, closing bracket, component length)

The accuracy dataset is used to calculate the performance and accuracy of the DCJ-RNA algorithm using different RNA structure sizes. This dataset consists of three pairs of RNA structures that are chosen from the GenBank [19] and Rfam database [20] and differ in size. The first pair of RNA structures consists of two small RNA structures; named D and E, as shown in Fig. 6.

**Fig. 7:**
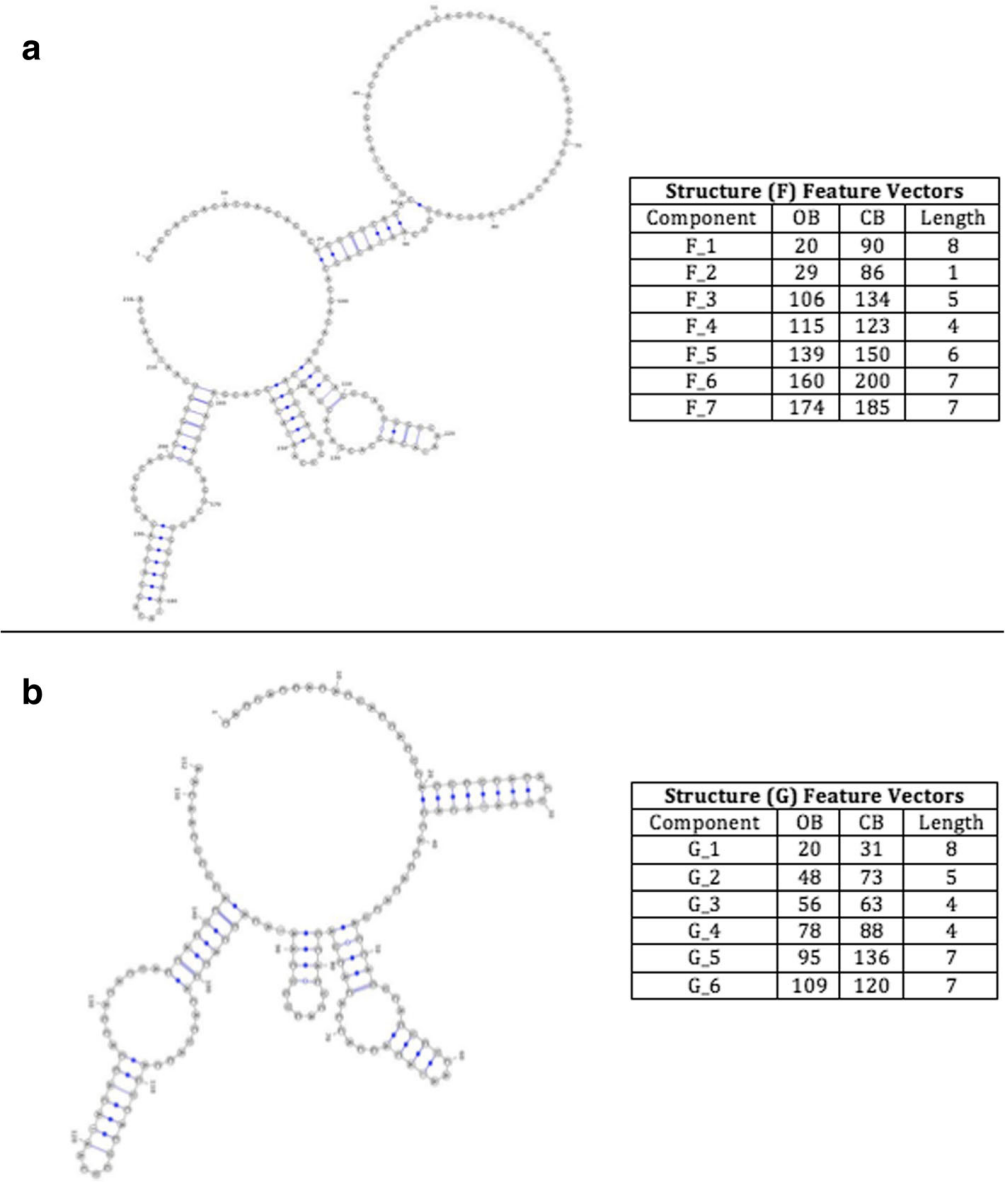
Structures F and G, respectively, with their features listed as follows (ComponentID, opening bracket, closing bracket, component length)

The second pair consists of two medium RNA structures; named F and G, as shown in Fig. 7.

**Fig. 8:**
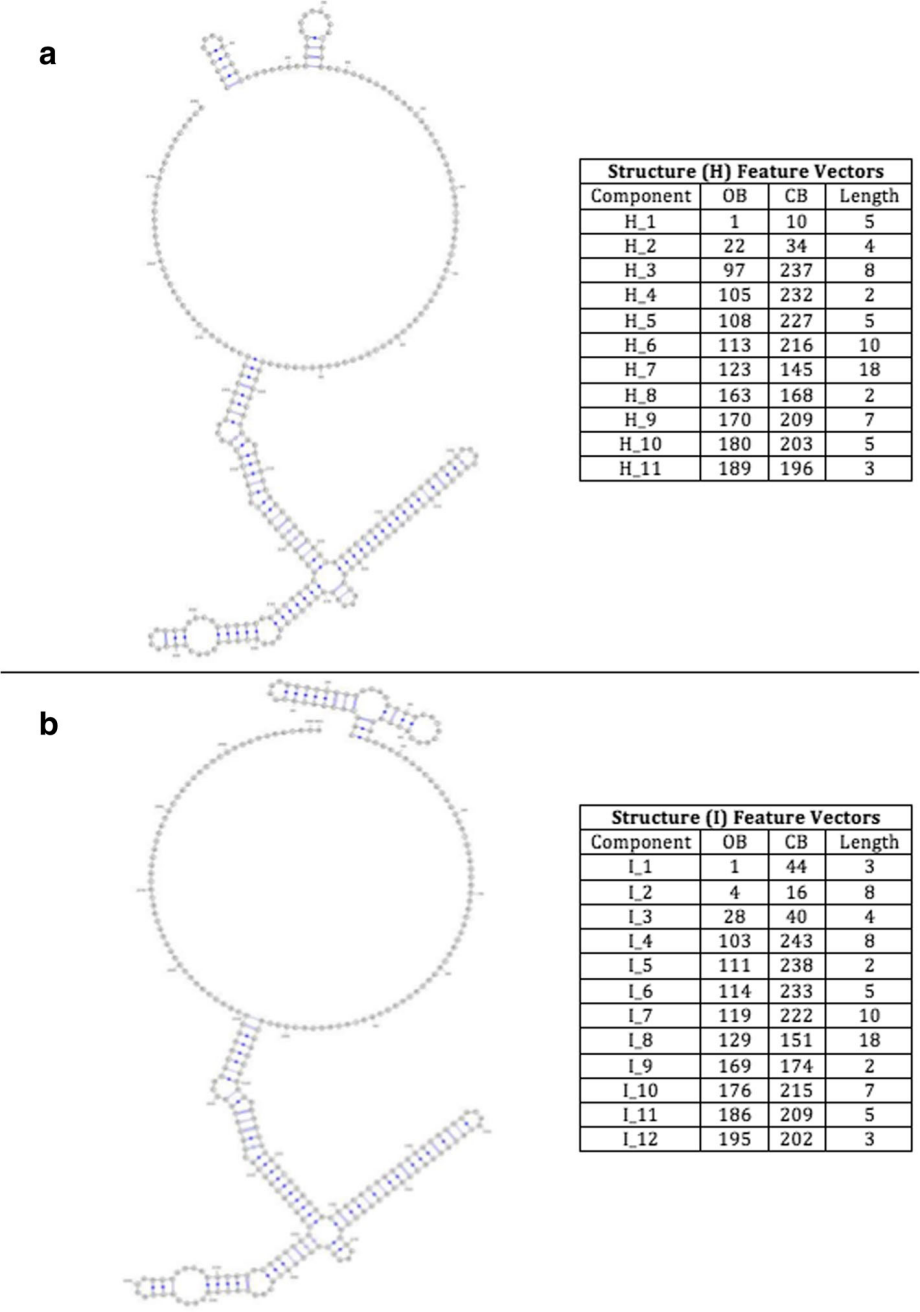
Structures H and I, respectively, with their features listed as follows (ComponentID, opening bracket, closing bracket, component length)

The third pair consists of two large RNA structures; named H and I, as shown in Fig. 8.

#### Scalability dataset

**Fig. 9:**
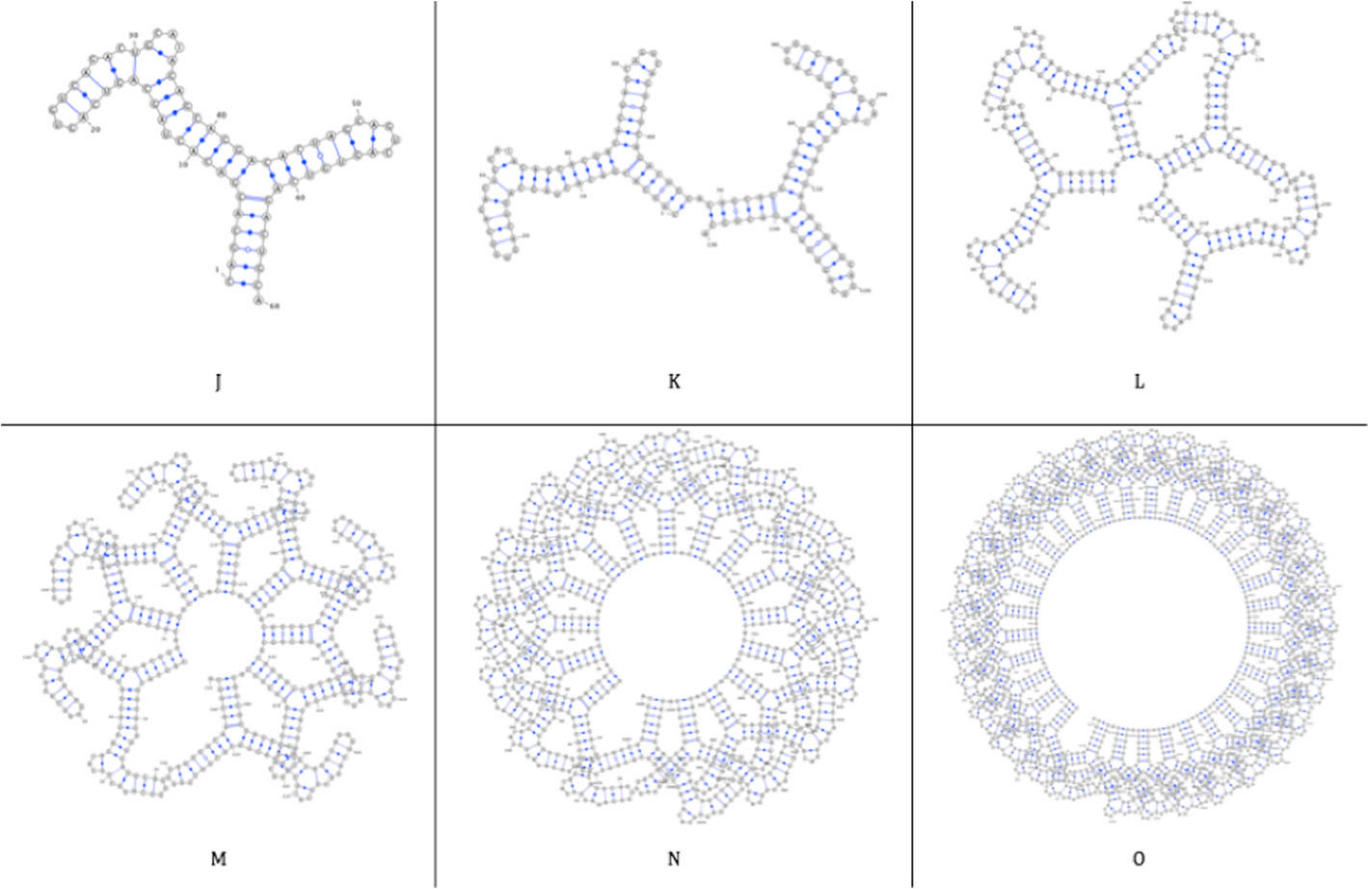
Scalability dataset with six RNA structures

The scalability dataset is used to calculate the scalability of the time and memory performance of the DCJ-RNA algorithm using different RNA structure sizes. This dataset consists of 11 RNA structures based on the first RNA structure, A, in the adjust dataset. Then the second structure is a duplicate of the first one, the third structure is a duplicate of the second one, and so on. The RNA structures’ numbers, names, sizes, and number of components are shown in Table 5. The first six RNA structures (J, K, L, M, N, and O) are shown in Fig. 9.

**Table 5:** RNA structures with their features

RNA structure #	1	2	3	4	5	6	7	8	9	10	11
RNA Structure Name	J	K	L	M	N	O	P	Q	R	S	T
Size (length)	68	136	272	544	1088	2176	4352	8704	10,336	20,672	41,344
Components Number	9	18	36	72	144	288	576	1152	1368	2736	5472

### Experiments

Three experiments are conducted - threshold adjustment, performance accuracy, and time and memory performance experiments, the experiments are obtained using real and simulated data in [19].

#### Threshold adjustment experiment

Threshold adjustment experiments are conducted to determine the best threshold enhancement (ε) value that gives the minimum number of rearrangement operations to make the RNA structures exactly the same or approximately similar.

##### Experiment setup

The used dataset is the adjust dataset, while fixed parameters are W_P_ equals 0 and W_cl_ and W_sl_ equal 1. Experiments are conducted for 10 values of threshold enhancement (ε) from 0 to 1.

##### Experiment results

We change the value of the threshold enhancement (ε) from 0.0, 0.1, 0.2, … 1.0 and obtain the result shown in Table 6 for both cases - similar structures with dissimilar sequences and similar structures with dissimilar sequences. As illustrated in Table 7, when the threshold enhancement (ε) equals 1.0, it means that the RNA structures are exactly similar but the number of the rearrangement operations is greater than the other values. On the other side, when threshold enhancement (ε) equals 0.0, it means that when the desired structure has less than or equal number of components as compared to the given structure, the order of the components is changed, and no components are added or deleted.

**Table 6:** Different threshold enhancement (ε) values with algorithm accuracy

	Similar structures and dissimilar sequences (35%)		Similar sequences and dissimilar structures (20%)	
Threshold enhancement (ε)	CompPSA	Rearrangement operations	CompPSA	Rearrangement operations
0.0	64%	2	59%	13
0.1	64%	2	71%	14
0.2	64%	2	71%	14
0.3	64%	2	71%	14
0.4	64%	2	71%	14
0.5	64%	2	94%	14
0.6	64%	2	94%	14
0.7	69%	3	94%	14
0.8	69%	3	97%	14
0.9	71%	4	100%	14
1.0	100%	7	100%	14

**Table 7:** Length similarity of small pairs of RNA structures in terms of component length and stem length

	E_1_	E_2_	E_3_	E_4_
D_1_	0.97	0.65	0.39	0.22
D_2_	0.5	0.74	0.35	0.21
D_3_	0.21	0.29	0.61	0.95

**Fig. 10:**
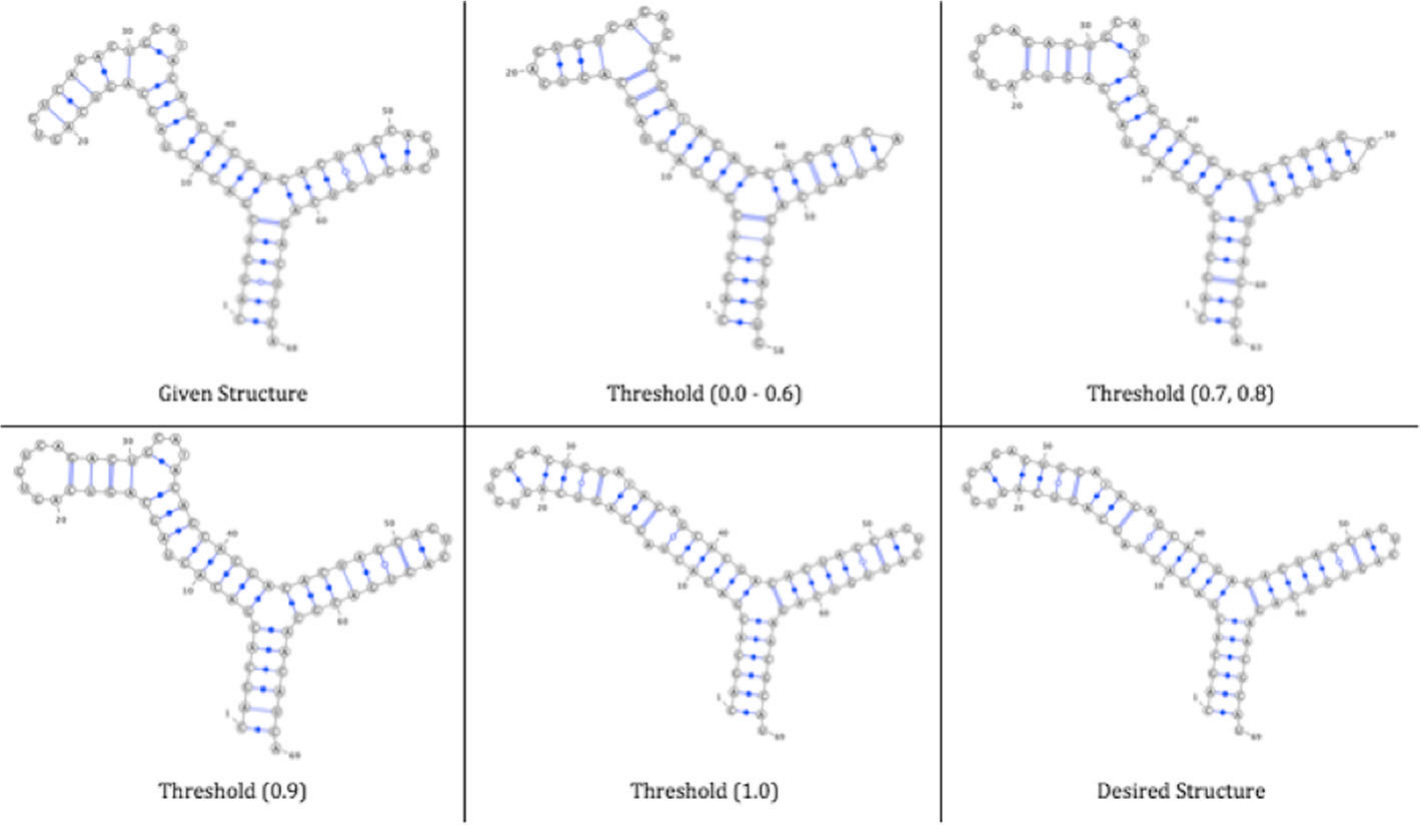
RNA structures after sorting for each threshold enhancement (ε)

From results, it can be seen that when the structures are similar, the best threshold enhancement (ε) equals 0.6, because of the similarity between structures and the number of rearrangement operations is reasonable; the structures after sorting for each threshold enhancement (ε) are shown in Fig. 10. For the same reason, when the structures are dissimilar, the best threshold enhancement (ε) equals 0.8.

#### Performance accuracy experiment

The performance accuracy experiment is conducted to show the accuracy of the DCJ-RNA algorithm with different RNA sizes. To test the effect of the DCJ-RNA algorithm and calculate the similarity between structures, the CompPSA algorithm [6] is used.

##### Experiment setup

The dataset used is accuracy dataset. Since all three RNA structures pairs are similar in their structures and dissimilar in their sequences, the threshold enhancement (ε) equals 0.6 and fixed parameters are W_P_ equals 0 and W_cl_ and W_sl_ are equal to 1.

##### Experiment results

DCJ-RNA was applied to three pairs of RNA structures - small, medium, and large RNA structures. Each experiment is discussed in detail in the following.

#### Small pairs of RNA structures

- **Step 1** - Alignment of Similar Components Based on Component Lengths and Stem Lengths

Calculate the similarity between components as shown in Table 8. Then assign similar components together whenever the similarity between them is greater than or equal to threshold enhancement (ε), which is 0.6. Here, assign D_1_ with E_1_, E_4_ with D_3_, E_2_ with D_2_, and add E_3_.

- **Step 2 -** Permutation Generation

**Table 8:** SortArray for small pairs of RNA structures

Index	0	1	2	3	4
SortArray[0]	3	1(D_1_)	2(D_2_)	6(E_3_)	3(D_3_)
SortArray[1]	0				
SortArray[2]	1	6(E_3_)			

Construct SortArray, fill it as shown in Table 9. After that, construct the permutations to apply the DCJ algorithm.

- **Step 3 -** Apply the Double Cut and Join Algorithm

**Table 9:** Length similarity of medium pairs of RNA structures in terms of component length and stem length

	G_1_	G_2_	G_3_	G_4_	G_5_	G_6_
F_1_	0.39	0.43	0.16	0.2	0.71	0.35
F_2_	0.11	0.23	0.13	0.16	0.23	0.12
F_3_	0.56	0.95	0.44	0.53	0.68	0.59
F_4_	0.52	0.51	0.96	0.92	0.29	0.58
F_5_	0.81	0.66	0.63	0.72	0.48	0.9
F_6_	0.54	0.65	0.28	0.33	0.99	0.55
F_7_	0.91	0.62	0.55	0.64	0.55	1.0

Construct the permutations to apply the DCJ algorithm. First permutation is (chr_1_ = {1,2,3} and chr_2_ = {6}). (Note - permutation represented as a sequence of numbers, to differentiate between the first structure’s components and the second structure’s components, we represent the second structure’s component i as i + N, where N equals the number of components in the first structure.) The second permutation is - (chr_1_ = {1,2,6,3} and chr_2_ = {}). Represent each genome with its adjacencies and telomeres to apply the DCJ algorithm, the first and second permutations are as follows:

- The first permutation is: {{1 t}, {1 h, 2 t}, {2 h, 3 t}, {3 h}, {6 t}, {6 h}}
- The second permutation is: {{1 t}, {1 h, 2 t}, {2 h, 6 t}, {6 h, 3 t}, {3 h}}

After applying the DCJ algorithm, we obtain the number of the DCJ operations, which is 2, and the sorting scenario is:

- {{{1 t}, {1 h, 2 t}, {2 h, 3 t}, {3 h}, {6 t}, {6 h}}, {{1 t}, {1 h, 2 t}, {2 h, 6 t}, {6 h, 3 t}, {3 h}}}

**Fig. 11:**
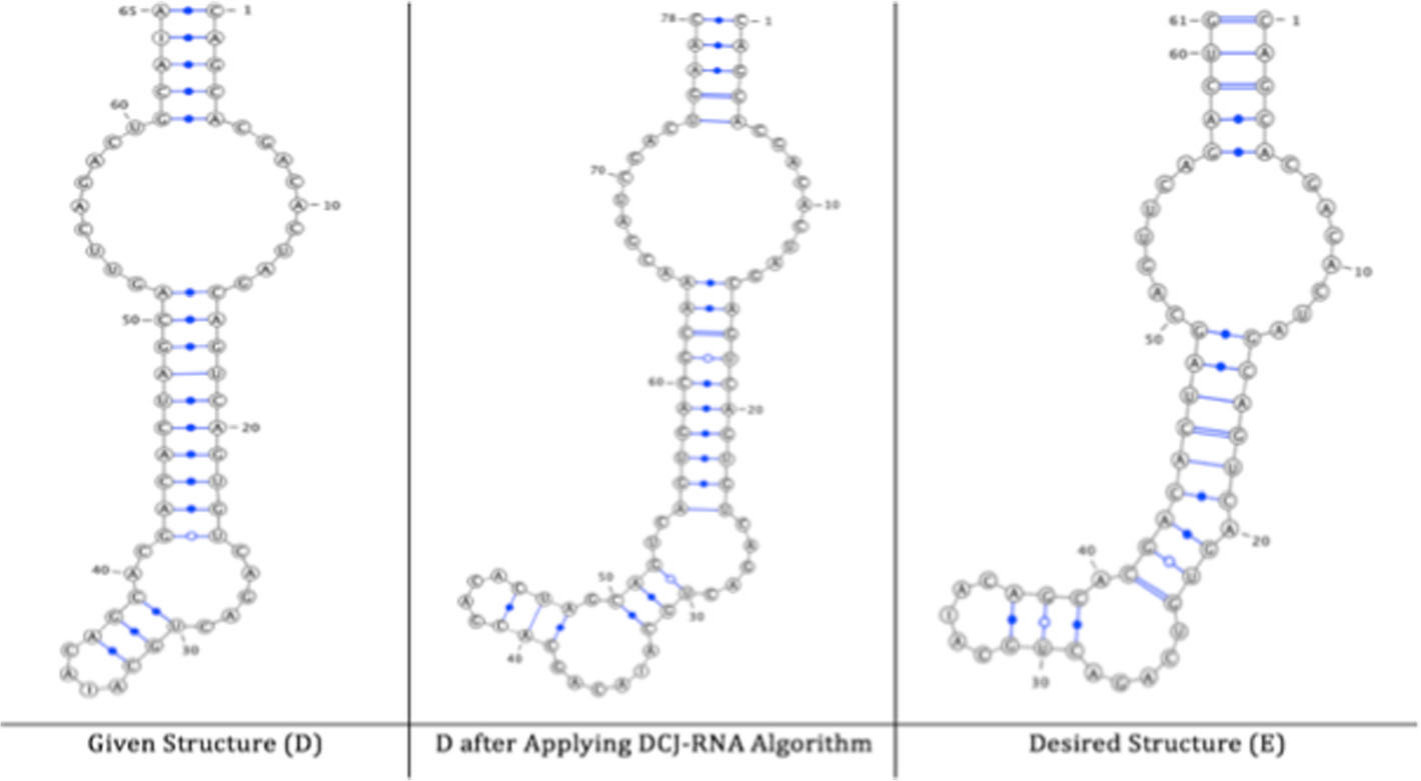
Given, sorted, and desired structures for small pairs of RNA structures

The similarity between the given structures D and E is 58% before applying any changes, while it increases to 85% after applying the DCJ-RNA algorithm; see Fig. 11.

#### Medium pairs of RNA structures

- **Step 1** - Alignment of Similar Components Based on Component Lengths and Stem Lengths

Calculate the similarity between components as shown in Table 10, then, assign F_7_ with G_6_, F_6_ with G_5_, F_4_ with G_3_, F_3_ with G_2_, F_5_ with G_1_, delete F_1_, delete F_2,_ and add G_4_.

- **Step 2 -** Permutation Generation

**Table 10:** SortArray for medium pairs of RNA structures

Index	0	1	2	3	4	5	6	7
SortArray[0]	7	5(F_5_)	3(F_3_)	4(F_4_)	11(G_4_)	6(F_6_)	7(F_7_)	
SortArray[1]	2	1(F_1_)	2(F_2_)					
SortArray[2]	1	11(G_4_)						

Construct SortArray, fill it as shown in Table 11. After that, construct the permutations to apply the DCJ algorithm.

- **Step 3 -** Apply the Double Cut and Join Algorithm

**Table 11:** Length similarity of large pairs of RNA structures in terms of component length and stem length

	I_1_	I_2_	I_3_	I_4_	I_5_	I_6_	I_7_	I_8_	I_9_	I_10_	I_11_	I_12_
H_1_	0.35	0.63	0.83	0.13	0.11	0.2	0.15	0.23	0.38	0.39	0.67	0.62
H_2_	0.44	0.59	1.0	0.13	0.15	0.2	0.14	0.21	0.41	0.38	0.65	0.66
H_3_	0.26	0.24	0.13	1.0	0.37	0.7	0.77	0.26	0.04	0.44	0.24	0.07
H_4_	0.42	0.11	0.15	0.37	1.0	0.56	0.31	0.09	0.1	0.23	0.2	0.12
H_5_	0.41	0.21	0.2	0.7	0.56	1.0	0.64	0.21	0.06	0.45	0.37	0.11
H_6_	0.27	0.27	0.14	0.77	0.31	0.64	1.0	0.37	0.04	0.48	0.26	0.08
H_7_	0.27	0.41	0.21	0.26	0.09	0.21	0.37	1.0	0.06	0.52	0.36	0.11
H_8_	0.21	0.21	0.41	0.04	0.1	0.06	0.04	0.06	1.0	0.12	0.23	0.66
H_9_	0.6	0.57	0.38	0.44	0.23	0.45	0.48	0.52	0.12	1.0	0.63	0.21
H_10_	0.57	0.64	0.65	0.24	0.2	0.37	0.26	0.36	0.23	0.63	1.0	0.39
H_11_	0.36	0.36	0.66	0.07	0.12	0.11	0.08	0.11	0.66	0.21	0.39	1.0

Construct the permutations to apply the DCJ algorithm. The first permutation is (chr_1_ = {1, 2, 3, 4, 5, 6, 7} and chr_2_ = {11}). The second permutation is - (chr_1_ = {5, 3, 4, 11, 6, 7} and chr_2_ = {1, 2}). Represent each genome with its adjacencies and telomeres as:

- The first permutation is: {{1 t}, {1 h, 2 t}, {2 h, 3 t}, {3 h, 4 t}, {4 h}, {5 t}, {5 h, 6 t}, {6 h, 7 t}, {7 h}, {11 t}, {11 h}}
- The second permutation is: {{5 t}, {5 h, 3 t}, {3 h, 4 t}, {4 h, 11 t}, {11 h, 6 t}, {6 h, 7 t}, {7 h}, {1 t}, {1 h, 2 t}, {2 h}}

After applying the DCJ algorithm, we obtain the number of the DCJ operations, which is 4, and the sorting scenario is:

- {{{1 t}, {1 h, 2 t}, {2 h, 3 t}, {3 h, 4 t}, {4 h}, {5 t}, {5 h, 6 t}, {6 h, 7 t}, {7 h}, {11 t}, {11 h}},
- {{1 t}, {1 h, 2 t}, {2 h, 6 t}, {3 h, 4 t}, {4 h}, {5 t}, {5 h, 3 t}, {6 h, 7 t}, {7 h}, {11 t}, {11 h}}
- {{1 t}, {1 h, 2 t}, {2 h, 6 t}, {3 h, 4 t}, {4 h, 11 t}, {5 t}, {5 h, 3 t}, {6 h, 7 t}, {7 h}, {11 h}}
- {{1 t}, {1 h, 2 t}, {2 h}, {3 h, 4 t}, {4 h, 11 t}, {5 t}, {5 h, 3 t}, {6 h, 7 t}, {7 h}, {11 h, 6 t}}}

**Fig. 12:**
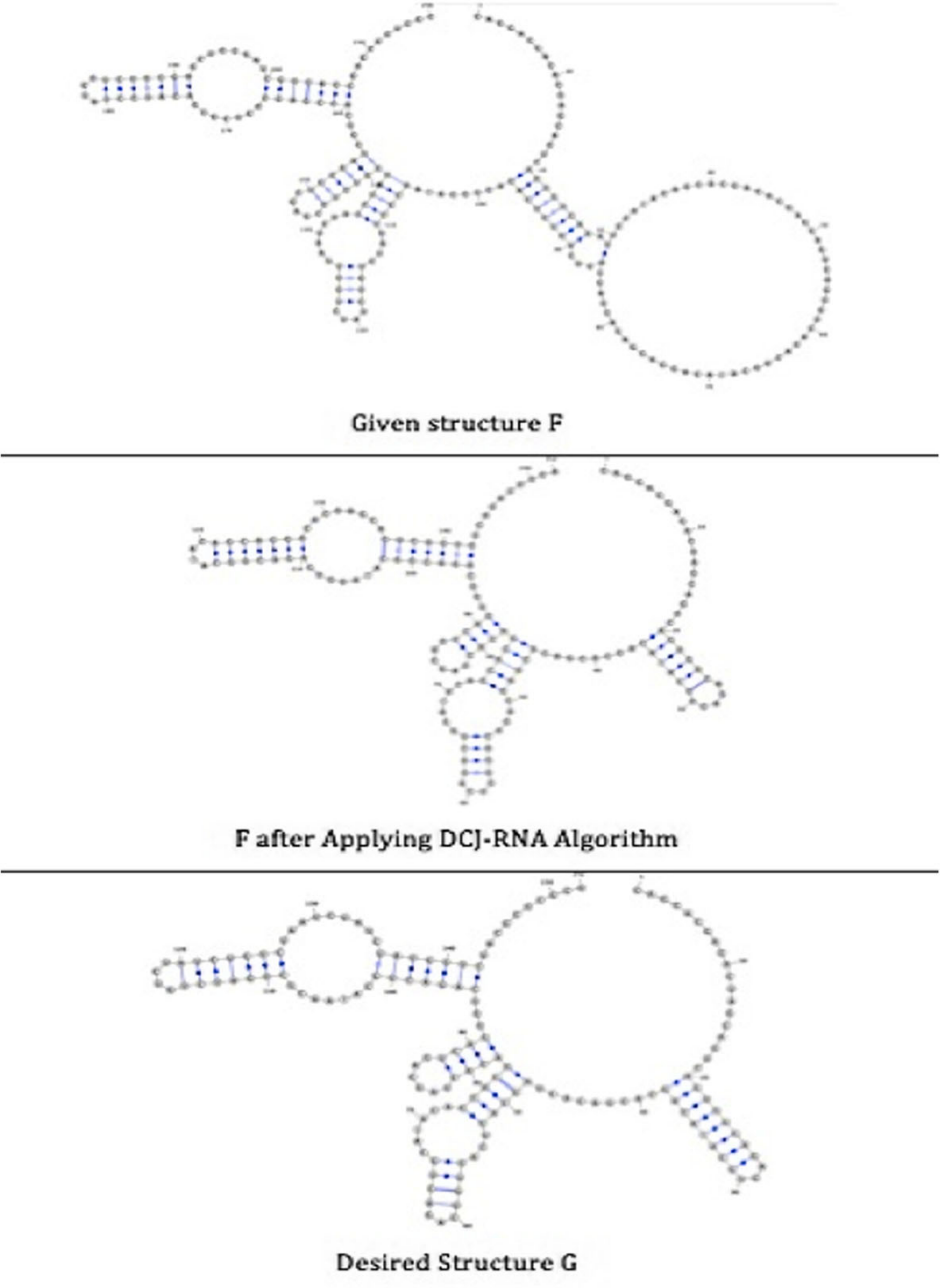
Given, sorted, and desired structures for medium pairs of RNA structures

The similarity between the given structures F and G is 49% before applying any changes, while it increases to 94% after applying the DCJ-RNA algorithm; see Fig. 12.

#### Large pairs of RNA structures

- **Step 1** - Alignment of Similar Components Based on Component Lengths and Stem Lengths

Calculate the similarity between components as shown in Table 4.7, then, assign H_1_ with I_2_, H_2_ with I_3_, H_3_ with I_4_, H_4_ with I_5_, H_5_ with I_6_, H_6_ with I_7_, H_7_ with I_8_, H_8_ with I_9_, H with I_10_, H_10_ with I_11_, H_11_ with I_12,_ and insert I_1_.

- **Step 2 -** Permutation Generation

Construct SortArray fill it as shown in Table 12. After that, construct the permutations to apply the DCJ algorithm.

- **Step 3 -** Apply the Double Cut and Join Algorithm

**Table 12:** SortArray for large pairs of RNA structures

Index	0	1	2	3	4	5	6	7	8	9	10	11	12
SortArray[0]	12	12(I_1_)	1(H_1_)	2(H_2_)	3(H_3_)	4(H_4_)	5(H_5_)	6(H_6_)	7(H_7_)	8(H_8_)	9(H_9_)	10(H_10_)	11(H_11_)
SortArray[1]	0												
SortArray[2]	1	12(I_1_)											

Construct the permutations to apply the DCJ algorithm. The first permutation is (chr_1_ = {1, 2, 3, 4, 5, 6, 7, 8, 9, 10, 11} and chr_2_ = {12}). The second permutation is - (chr_1_ = {12, 1, 2, 3, 4, 5, 6, 7, 8, 9, 10, 11} and chr_2_ = {}). Represent each genome with its adjacencies and telomeres to apply the DCJ algorithm, as the following:

- The first permutation is: {{1 t}, {1 h, 2 t}, {2 h, 3 t}, {3 h, 4 t}, {4 h, 5 t}, {5 h, 6 t}, {6 h, 7 t}, {7 h, 8 t}, {8 h, 9 t}, {9 h, 10 t}, {10 h, 11 t}, {11 h}, {12 t}, {12 h}}
- The second permutation is: {{12 t}, {12 h, 1 t}, {1 h, 2 t}, {2 h,3 t}, {3 h, 4 t}, {4 h, 5 t}, {5 h, 6 t}, {6 h, 7 t}, {7 h, 8 t}, {8 h, 9 t}, {9 h, 10 t}, {10 h, 11 t}, {11 h}}

After applying the DCJ operations, we get the number of the DCJ algorithm, which is 2, and the sorting scenario is:

- {{{12 t}, {1 t}, {1 h, 2 t}, {2 h, 3 t}, {3 h, 4 t}, {4 h, 5 t}, {5 h, 6 t}, {6 h, 7 t}, {7 h, 8 t}, {8 h, 9 t}, {9 h, 10 t}, {10 h, 11 t}, {11 h},{12 h}},
- {{12 t}, {12 h, 1 t}, {1 h, 2 t}, {2 h,3 t}, {3 h, 4 t}, {4 h, 5 t}, {5 h, 6 t}, {6 h, 7 t}, {7 h, 8 t}, {8 h, 9 t}, {9 h, 10 t}, {10 h, 11 t}, {11 h}}}

**Fig. 13:**
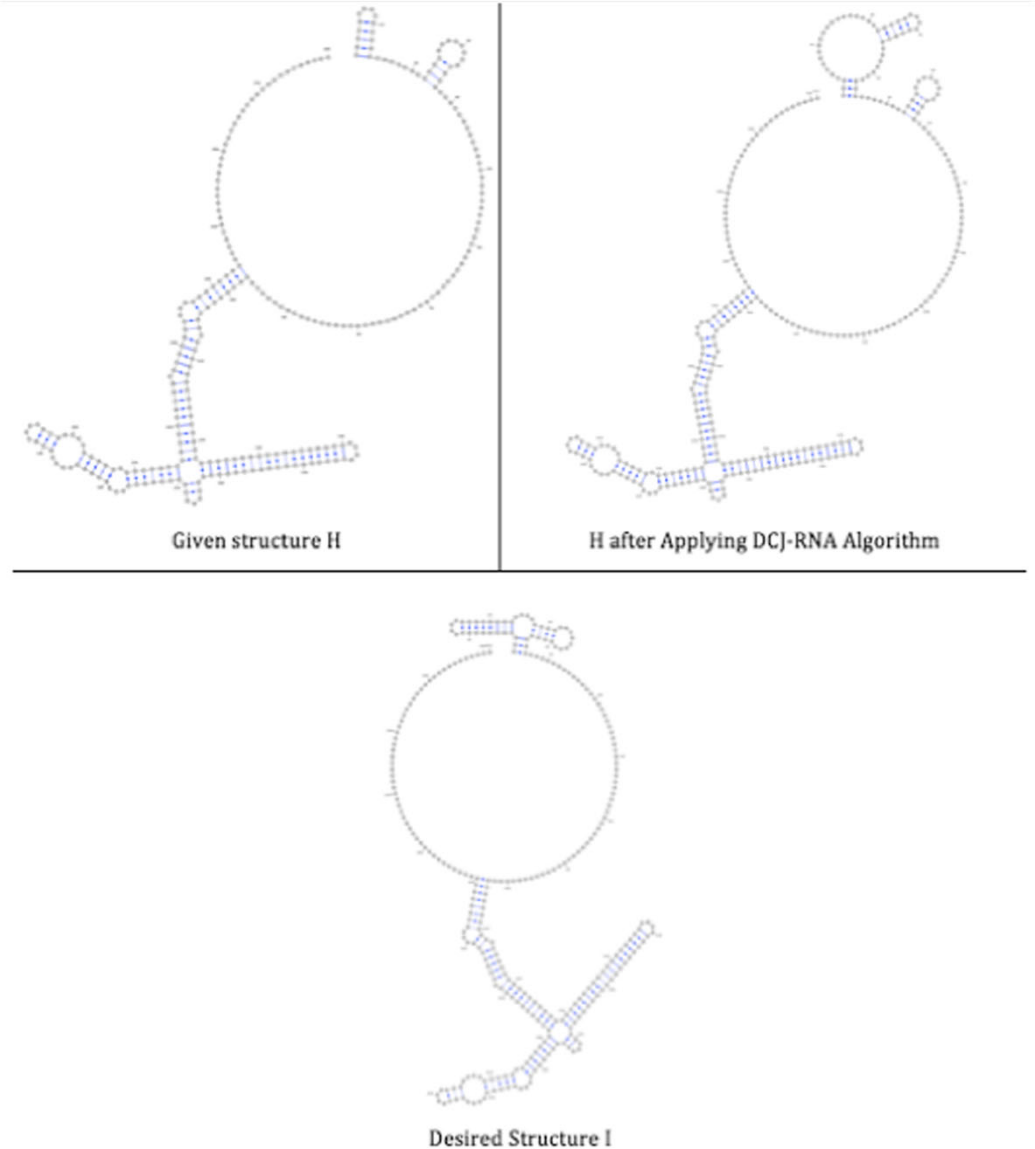
Given, sorted, and desired structures for large pairs of RNA structures

The similarity between the given structures H and I is 84% before applying any changes, while it increases to 91% after applying the DCJ-RNA algorithm; see Fig. 13.

#### Time & Memory performance experiment

The time and memory performance experiment is conducted to test the performance of the DCJ-RNA algorithm using different RNA structure sizes.

##### Experiment setup

The scalability dataset is used, while fixed parameters W_P_ equals 0 and W_cl_ and W_sl_ are equal to 1. Threshold enhancement (ε) equals 0.6 since structures are similar. The two structures in each experiment are identical which means the similarity between them is 100%.

##### Experiment results

**Fig. 14:**
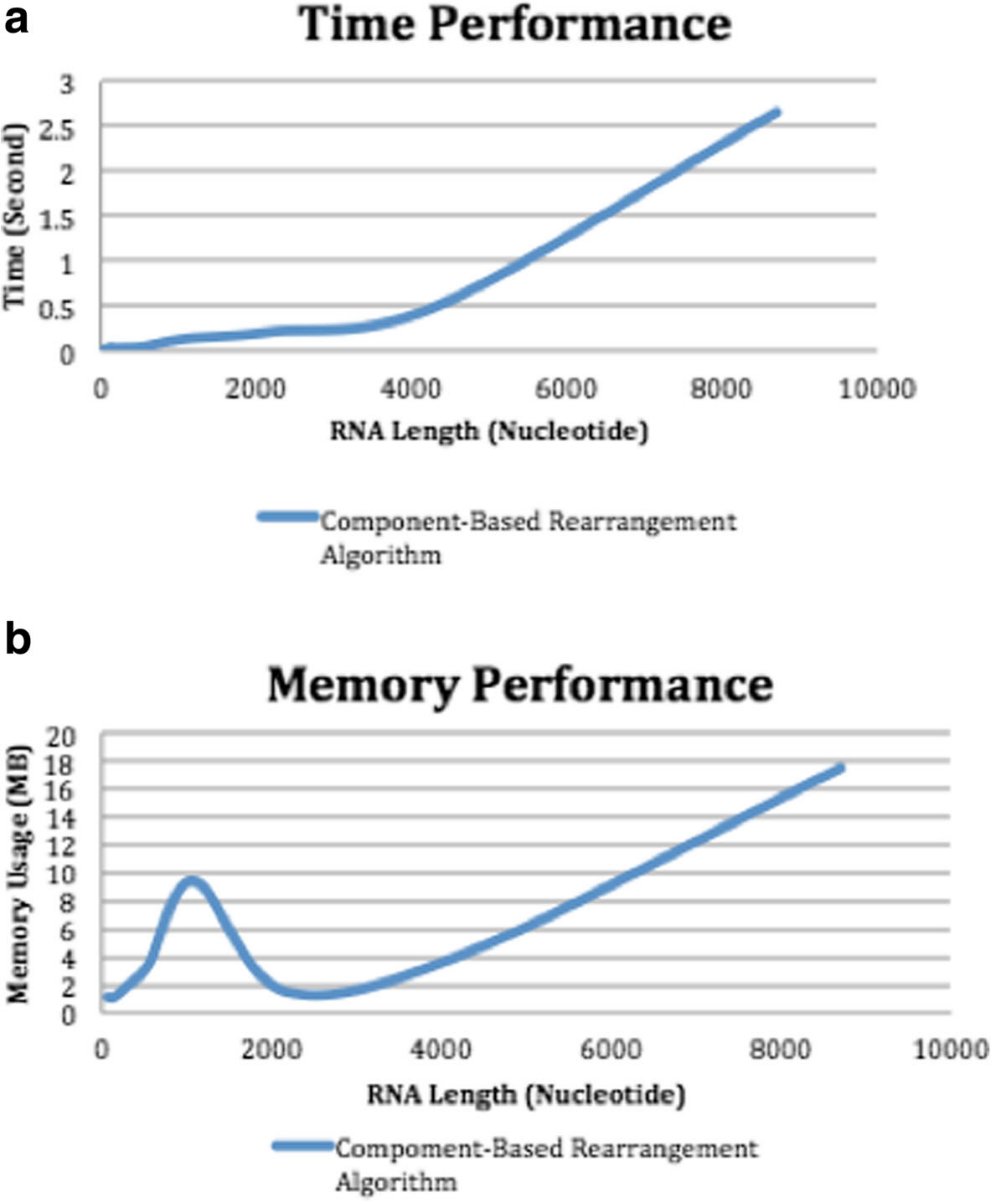
The performance results for time (**a**) and memory (**b**)

*Consider the maximum number of components to be N; the time complexity of step 1 is O(N log N) for the worst case. Each time we have to search for the maximum value for N values then discard the row and column related to maximum value, as a result, the next search is applied to (N-1) components and so on. The time complexity of the second step is O(N), since this step determines the inserted components and the deleted components. The algorithm moves through the entries only once to fill SortArray in which they are all of size N. For step three, the time complexity is O(N) since the DCJ algorithm is used. Therefore, the worst time for the entire algorithm is O(N log N).* Table 13 *and* Fig. 14 *confirm the time performance analysis empirically using the scalability dataset. The space requirement for the first step is O(N* ^*2*^ *) when the same number of components are present. For the second step, the memory takes O(3 N) for SortArray. For the third step, the space of memory is O(2 N). Hence, the total space requirement for DCJ-RNA algorithm is O(N* ^*2*^ *).* Table 13 *shows time and memory performance results from this experiment and the corresponding graph representation* (Fig. 14).

**Table 13:** Time and memory performance results of the DCJ-RNA algorithm

Length	Number of components	Time in seconds	Memory usage in MB
68	9	0.010739	1.11
136	18	0.020159	1.11
272	36	0.026246	1.78
544	72	0.039157	3.44
1088	144	0.130200	9.38
2176	288	0.208723	1.50
4352	576	0.502496	4.43
8704	1152	2.657500	17.50

## Conclusion

The DCJ-RNA algorithm is proposed and is able to describe the evolutionary scenarios that are based on rearrangements of secondary structures rather than sequences. The DCJ-RNA algorithm is optimal. Since RNA secondary structures reveal more functionality, this algorithm can help in the comparison between the functionality of structures. Real data is used to illustrate the details of the proposed algorithm. It demonstrates that the algorithm is able to detect the minimum number of rearrangement operations in order to make one structure more similar to the other. A rearrangement scenario increases similarity between the first structure and any other structure. This creates an ideal framework for applying rearrangement operations to secondary structures rather than sequences.

The algorithm is applied to non-interacting patterns only. Therefore, future work should extend the algorithm to consider interacting RNA patterns. In addition, the researchers would like to explore other well-defined structures, such as chemical structures, and investigate the application of a similar approach that can define a scenario for changing one structure into another structure. Using the DCJ-RNA approach, we would also like to develop a tool that can help biologists compare RNA structures to folded RNA structures that are based on the corresponding RNA sequence. This tool, which is unavailable, would be ideal for biologists, as suggested at the RECOMB-CG conference in 2014.
